# Total neoadjuvant chemoradiotherapy plus anti PD-1 for mid-to-low locally advanced rectal cancer: study protocol of a prospective, single arm, phase II study (STARS - RC06)

**DOI:** 10.3389/fonc.2025.1594927

**Published:** 2025-08-08

**Authors:** Meichen Gu, Yuchen Guo, Liang He, Xuan Sun, Tingting Liang, Zhuo Wang, Ying Li, Zheng Liu, Jingyu Wang, Xiang Qiu, Liang Guo, Pengyu Chang, Quan Wang

**Affiliations:** ^1^ Radiation Oncology and Therapy Department, First Hospital of Jilin University, Changchun, China; ^2^ Gastric and Colorectal Surgery Department, General Surgery Center, First Hospital of Jilin University, Changchun, China; ^3^ Oncology Department, First Hospital of Jilin University, Changchun, China; ^4^ Department of Radiology, First Hospital of Jilin University, Changchun, China; ^5^ Department of Pathology, First Hospital of Jilin University, Changchun, China

**Keywords:** locally advanced rectal cancer, total neoadjuvant therapy, immunotherapy, immune checkpoint inhibitor, pathological complete response

## Abstract

**Background:**

Neoadjuvant chemoradiotherapy has become the standard treatment for mid-to-low location LARC. Recently, total neoadjuvant therapy (TNT) has been used in patients with ‘high-risk’ or ‘very high-risk’ LARC according to ESMO guideline (2017). TNT not only increases the pathological complete response (pCR) rate, but also provides patients with more opportunities to preserve organ function. However, TNT mode seems to have reached a plateau. Some clinical studies published in recent years have also confirmed that different modes of neoadjuvant chemoradiotherapy combined with ICIs can further improve the pCR rate to varying degrees. It is necessary to explore an appropriate treatment mode for patients with mid-to-low location, who are unresectable or difficult to achieve R0 resection at the initial stage of surgery and have a strong desire to preserve anal cavity. Numerous basic researches have demonstrated that radiotherapy can remodel the tumor immune microenvironment and play a potential synergistic effect on ICIs. Therefore, this study will aim to explore whether TNT combined with ICIs could improve pCR rates in patients with ‘high-risk’ or ‘very high-risk’ LARC.

**Methods:**

This prospective, single-center, single-arm phase II trial aims to assess the pCR rate of neoadjuvant long-course concurrent chemoradiotherapy sequential 6 cycles CapeOX regimen combined with sintilimab in ‘high-risk/very high-risk’ LARC patients with mid-to-low location and with pMMR phenotype. The primary endpoint for this study is the pCR rate. Secondary endpoints include 2-year sustained of clinical complete response (cCR) rate; CR rate (the rate of patients with sustained cCR for 2 years and pCR); major pathological remission; neoadjuvant rectal score; 3-year non-regrowth disease free survival; 3-year disease-free survival; 3-year overall survival; 3-year localized recurrence-free survival; 3-year distant metastasis free survival; 3-year stoma-free survival, anal sphincter preservation rate; surgical R0 resection rate and safety (adverse events during neoadjuvant therapy and 30 days after surgery, as well as tolerance).

**Discussion:**

This study will investigate whether neoadjuvant long-course concurrent chemoradiotherapy sequential total neoadjuvant chemotherapy combined with immunotherapy could further enhances tumor pCR rate in ‘high-risk/very high-risk’ LARC patients with mid-to-low location and with pMMR phenotype and is expected to improve prognosis.

**Trial registration:**

ClinicalTrials.gov NCT05998122.

## Background

Among all cancers, colorectal cancer ranks third in incidence and second in mortality worldwide (1). In China, the incidence of rectal cancer is on the rise, and most patients are diagnosed with locally advanced rectal cancer (LARC) at initial diagnosis (2), characterized by low rates of R0 resection and anal preservation (3). Preoperative neoadjuvant chemoradiotherapy (nCRT) has emerged as the standard treatment for mid-to-low location LARC (4). Statistical data indicates that nCRT achieves pathological complete response (pCR) rate of 8% - 24%, offering patients who achieved clinical complete response (cCR) potential ‘Watch and Wait’ opportunities (5–8). Preserving anal sphincter function while maintaining efficacy has become a key issue for LARC. Clinical trials demonstrate that total neoadjuvant therapy (TNT) can improve both pCR rates and organ preservation. However, in most trials, the pCR rate related to TNT was still lower than 30%, highlighting the need for novel strategies to enhance tumor response (9). The European Society for Medical Oncology (ESMO) guideline classify patients into different subgroups based on digital rectal examination (DRE), rectal high-resolution magnetic resonance imaging (HRMRI), and rectal intraluminal ultrasound (10). The guideline recommends adjusting treatment regimens for patients in different risk levels to achieve better efficacy and lower risk in precise treatment. While PD-1/PD-L1 inhibitors revolutionized care of colorectal cancer with deficient mismatch repair (dMMR) or microsatellite instability-high (MSI-H) phenotype, these represent only 8% - 10% of the cases. The remaining cases of proficient mismatch repair (pMMR) or microsatellite stabilization (MSS) phenotype could hardly benefit from immune checkpoint inhibitors (ICIs) treatment. Interestingly, it has been demonstrated that radiotherapy has the effect of stimulating the release of neoantigens and activating immunity (11). Building on this, the VOLTAGE study demonstrated that adding ICIs after nCRT could increase the pCR rate of MSS phenotype rectal cancer to 30% (12). At present, studies have shown that TNT combined with immunotherapy demonstrates better tumor regression and pCR rates, but its survival benefits such as DFS and OS need longer follow-up (13). Crucially, no solid evidence yet confirms whether adding immunotherapy significantly boosts pCR rates for mid-to-low location LARC patients with ‘high-risk/very high-risk’ according to ESMO guideline. Our center conducted a prospective, single-arm, observational study of long course concurrent chemoradiotherapy consolidation chemotherapy combined with PD-1 inhibitors, and the results are being submitted (STARS-RC03, NCT04906044). A total of 29 pMMR LARC patients received neoadjuvant long-course concurrent chemoradiotherapy followed by at least 3 cycles of CapeOX chemotherapy combined with PD-1 inhibitor. One patient discontinued treatment and was not effectively evaluated for efficacy. Our results showed that this regimen was safe and tolerated. Grade III toxicity was mainly associated with chemotherapy-induced hematological toxicity (51.71%, 15/29), and was reversed through active symptomatic treatment. No grade III or higher immunotherapy-related toxicity was observed. The pCR rate was 17.86% (5/28), and the overall complete response rate (cCR and pCR) was 50% (14/28). In 19 patients who undergone radical surgery, the major pathological remission (MPR) rate was 73.58% (14/19), and the R0 resection rate was 89.47% (17/19). Subgroup analysis found that the CR rate was positively correlated with the number of consolidation therapy, but the incidence of grades III or higher adverse events did not increase with the number of consolidation therapy. Building on these results, we propose the hypothesis that neoadjuvant long-course concurrent chemoradiotherapy sequential 6 cycles CapeOX regimen combined with PD-1 inhibitor further enhances the tumor pCR rate and has the potential to improve prognosis in mid-to-low location LARC patients with ‘high-risk/very high-risk’ and with pMMR phenotype.

## Methods and analysis

### Trial organization, ethical approval, and drug supply

The trial is co-sponsored by the Department of Gastric and Colorectal Surgery and the Department of Radiotherapy of the First Hospital of Jilin University. Scientific review was obtained from the Review Board of the First Hospital of Jilin University on August 16, 2023 (2023-HS-084). Ethical approval was obtained from the Ethics Committee (Approval No.23K162-002) of the First Hospital of Jilin University on October 18, 2023. All relevant materials have been registered on the official ClinicalTrials.gov website (NCT05998122). The PD-1 inhibitor selected for this study is sintilimab from Innovent Biologics, Inc.

### Study population

All included patients required pathological immunohistochemical confirmation of rectal adenocarcinoma with intact expressions of all 4 MMR proteins, had mid-to-low location tumors (distal margin ≤10 cm from anal verge) verified by HIMRI, and were risk-stratified according to ESMO rectal cancer guidelines (2017) as ‘high-risk’ (cT3c/d or very low localization levators threatening, MRF-negative cT3c/d mid-rectum, cN1-N2 with extranodal involvement, EMVI-positive, or limited cT4aN0) or ‘very high-risk’ (cT3 with MRF involved, any cT4a/b, or lateral lymph node metastasis) (10). Participants willingly volunteered for this clinical study and provided written informed consent. Detailed inclusion and exclusion criteria are available in **Table 1**. The study commenced in October 2023 and is expected to end before December 2028. If all enrolled patients complete all treatment plans during this period, the study will be terminated. Study enrollment is ongoing, with the first patient recruited on October 31, 2023.

**Table 1:** Patient inclusion and exclusion criteria.

Inclusion criteria:	Exclusion criteria:
1. The patients and their families are able to understand and are willing to participate in this clinical study, and sign an informed consent form; 2. Age: 18~75 years old, no gender limit; 3. Pathologically diagnosed rectal adenocarcinoma: differentiated into Grade 1-3, that is, high, medium, and poorly differentiated tubular adenocarcinoma; classified as pMMR; 4. The initial risk stratification (from Rectal cancer: ESMO Clinical Practice Guidelines, 2017 edition) is as follows: 1) ‘high-risk (Locally Advanced Disease)’: cT3c/d or very low localization levators threatened, MRF clear; cT3c/d mid-rectum, cN1–N2 (extranodal), EMVI+, limited cT4aN0; 2) ‘very high-risk (Advanced disease)’: cT3 with any MRF involved, any cT4a/b, lateral node+; 5. The lower margin of the tumor located within 10cm from the anal margin (including patients with mrLR3 or mrLR4 at initial evaluation); 6. No distant metastasis; 7. ECOG PS score 0-1; 8. Hepatitis B Surface Antigen (HBsAg) (-) and Hepatitis B Core Antibody (HBcAb) (-). If HBsAg (+) or HBcAb (+), hepatitis B virus deoxyribonucleic acid (HBV-DNA) must be less than 1000 copies/mL or 200 IU/mL before entering the group; 9. HCV antibody (-); 10. The main organ function is normal; 11. No history of pelvic radiotherapy; 12. No history of rectal cancer surgery or chemotherapy; 13. Not accompanied by systemic infections requiring antibiotic treatment; 14. Heart, lung, liver, and kidney functions can tolerate surgery; 15. Others, based on the results of previous medical history, vital signs, physical examination or laboratory examination, the research doctor judges that you are suitable for participating in this clinical study.	1. Recurrent rectal cancer; 2. ECOG PS score 2-5; 3. Patients who are planning to undergo or have previously received organ or bone marrow transplantation; 4. Myocardial infarction or poorly controlled arrhythmia (including QTc interval ≥ 450 ms for males and ≥ 470 ms for females) occurred within 6 months before the first medication (QTc interval is calculated by Fridericia formula); 5. Existence of NYHA standard grade III to IV cardiac insufficiency or color Doppler ultrasound examination: LVEF (left ventricular ejection fraction) <50%; 6. Human immunodeficiency virus (HIV) infection; 7. Suffer from active tuberculosis; 8. Past and present patients with interstitial pneumonia, pneumoconiosis, radiation pneumonia, drug-related pneumonia, severely impaired lung function, etc., which may interfere with the detection and treatment of suspected drug-related lung toxicity; 9. Patients with active or suspicious autoimmune disease, or with a history of that; 10. Received treatment with live vaccines within 28 days before the first administration; except for inactivated viral vaccines for seasonal influenza; 11. Have received other antibody/drug treatments against immune checkpoints in the past, such as PD-1, PD-L1, CTLA4, etc.; 12. Known to have a history of severe allergies to any monoclonal antibody or research drug excipients; 13. Patients who have had a malignancy within the past 5 years with a survival rate significantly lower than the Center’s historical data on rectal cancer survival rates (properly treated basal cell carcinoma, squamous skin carcinoma, mini-kidney carcinoma, breast carcinoma, and papillary thyroid carcinoma are not included in this category); 14. The patient has had arterial embolism diseases in the past 6 months, such as angina pectoris, MI, TIA, CVA, etc.; 15. Have received other types of anti-tumor or experimental treatments; 16. The patient is a female during pregnancy or lactation; 17. The patient has other diseases or abnormal mental states, which may affect the patient’s participation in this study; 18. There are patients who may increase the risk of participating in research and research medication, or other severe, acute and chronic diseases, who are not suitable for clinical research based on the judgment of the investigator.

MRF, mesorectal fasciae; EMVI, extramural vascular invasion; ECOG PS score, Eastern Cooperative Oncology Group Performance Status; MI, myocardial infarction; TIA, transient ischemic attack; CVA, Cerebral Vascular Accident.

### Study design

**Figure 1:**
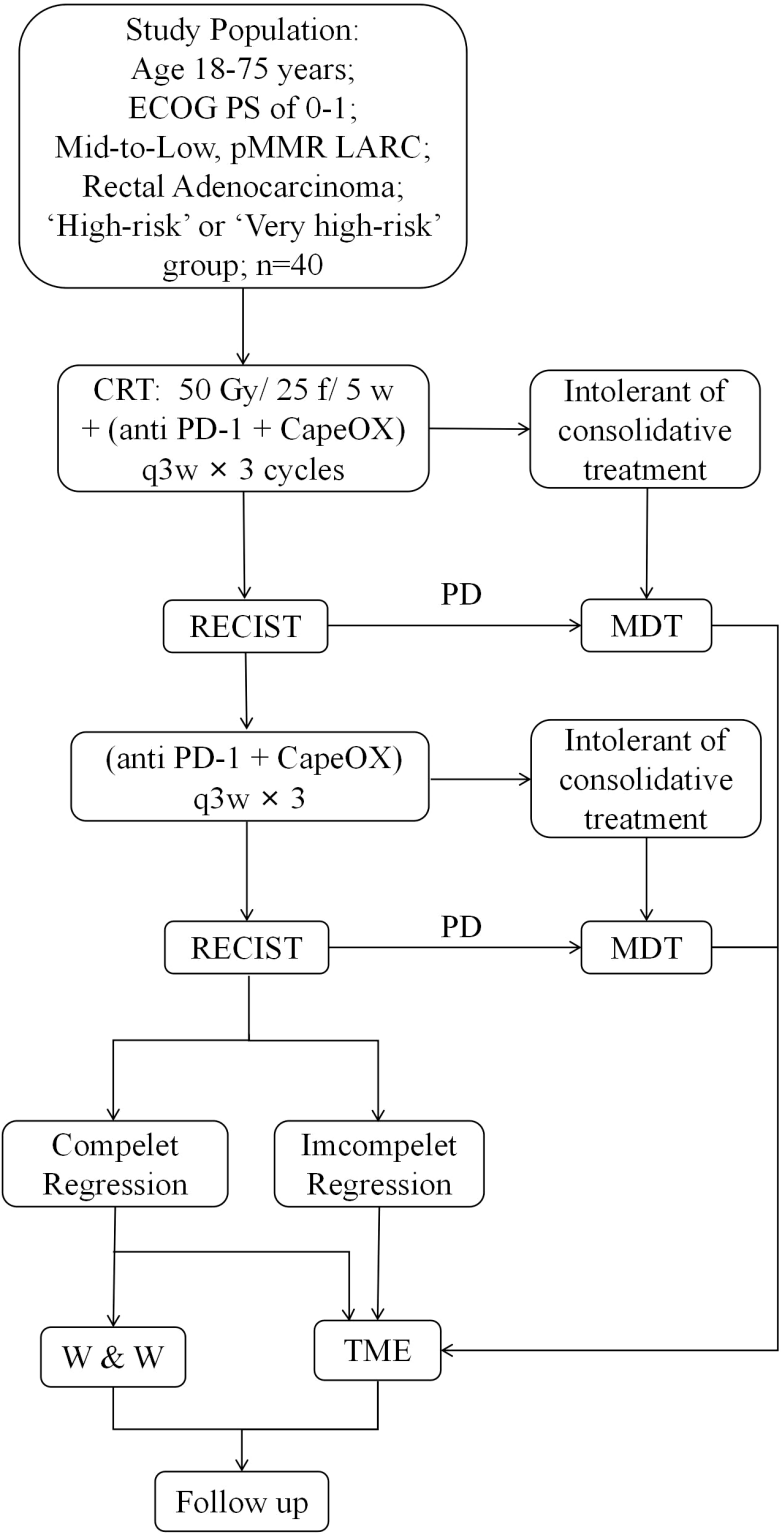
Study design. Recruitment pathway. All patients who are not eligible the STARS-RC06 trial will progress through these steps. pMMR, proficient mismatch repair. MDT, multidisciplinary team.

This prospective, single-center, single-arm phase II trial aims to assess the pCR rate of neoadjuvant long-course concurrent chemoradiotherapy sequential 6 cycles CapeOX regimen combined with sintilimab in mid-to-low location LARC with ‘high-risk/very-high-risk’ and with pMMR phenotype. The treatment process is showed in **Figure 1**. Enrolled patients will initially undergo long-course concurrent chemoradiotherapy, targeting the primary tumor area and regional lymphatic drainage area: 50 Gy/25 fractions/5 weeks with intensity-modulated radiation therapy or volumetric modulated arc therapy, alongside simultaneous capecitabine 825 mg/m^2^, twice daily, throughout the radiotherapy period. Following the completion of neoadjuvant concurrent chemoradiotherapy (7–10 days later), the subjects will receive 6 cycles of CapeOX combined with sintilimab treatment: sintilimab 200 mg intravenously on the day 1, oxaliplatin 130 mg/m2 intravenously on the day 1, and capecitabine 1000 mg/m^2^ orally twice daily from day 1 to day 14. Efficacy evaluations will occur every three cycles using DRE, HRMRI and endoscopy. Patients with progressive disease (PD) will undergo multidisciplinary team (MDT) discussion for alternative treatment strategies, while those with tumor regression and good tolerance will continue therapy. Patients whose tumors have not completely regressed after 6 cycles of consolidation treatment will directly receive surgical treatment. For patients evaluated as cCR, ‘Watch and Wait’ or surgery can be chosen based on MDT discussion and patient preference. The evaluation criteria of cCR according to ESMO guideline (2017), including no palpable tumor on DRE, no residual tumor or white scar under endoscopy, negative scar biopsy, and no residual tumor or residual fibrosis or residual wall thickening because of edema on HRMRI, and no suspicious lymph nodes on T2-weighted images (10, 14).

**Figure 2:**
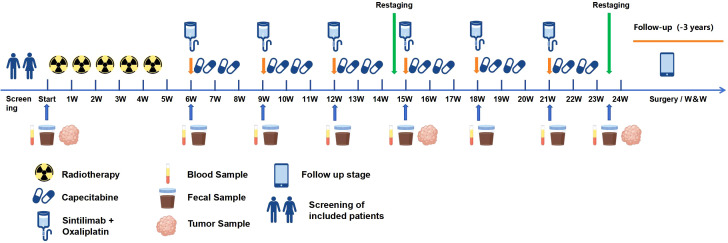
Sample collection procedure. Blood and fecal samples will be used for next generation sequencing (NGS), and tumor samples will be used for immunohistochemical staining and NGS.

Additionally, we will collect biological samples from all participants during treatment, The time points are shown in **Figure 2**. Fecal samples will be stored at -80°C for 16*S* rRNA sequencing. Tumor tissue samples will be used for the detection of PD-L1 expression level, MSI and TMB in the central laboratory. And blood samples will be used for MSI and TMB detection. Freshly collected specimens, resection, needle aspiration biopsy, punch or clamp biopsy will be all acceptable.

### Criteria for discontinuing or modifying interventions

If patients cannot tolerate consolidation therapy or experiences progression in efficacy evaluation, the subsequent treatment plan will be decided after discussion by the MDT and included in the follow-up. The specific treatment process is outlined in **Figure 1**. For grades III and IV chemotherapy-related adverse events (AEs), reduce the dose by 20% to 25%, as appropriate. All of immunotherapy-related adverse events (irAEs) were managed according to the NCCN Guidelines for Rectal Cancer (2022) for the management of immunotherapy toxicity (4). Drugs such as antihypertension, antidiabetes, painkillers and other symptomatic treatment are allowed. If a patient has hepatic injury or myelosuppression, hepatoprotectant, granulocyte colony-stimulating factor, and thrombopoietin may be used. The use of other anti-cancer drugs and traditional Chinese medicine is prohibited.

### Study endpoints and assessment

The primary endpoint of this trial is the pCR rate, defined as the percentage of patients who achieved pCR after nCRT in the eligible population. Secondary endpoints include 2-year sustained of cCR rate; CR rate (the rate of patients with sustained cCR for 2 years and pCR); MPR, which is defined as residual viable tumor (RVT) ≤10%; neoadjuvant rectal (NAR) score, which were calculated based on pathological and clinical staging (NAR=[5pN - 3(cT - pT) + 12]^2^/9.61); 3-year non⁃regrowth disease free survival; 3-year disease-free survival; 3-year overall survival; 3-year localized recurrence-free survival; 3-year distant metastasis free survival; 3-year stoma-free survival, anal sphincter preservation rate; surgical R0 resection rate and safety (adverse events during neoadjuvant therapy and 30 days after surgery, as well as tolerance). In addition, the study also has exploratory endpoints to further investigate potential factors related to antitumor therapy, such as infiltrating lymphocytes in tumor tissue, the structure of gut microbiome, and RAS/BRAF status.

The per-protocol set (PP) is defined as subjects who strictly adhered to the study protocol without major violations, completed all treatment and follow-up, and had measurable data for the primary endpoint (pCR). The modified intention-to-treat set (mITT) excludes participants who received no intervention or provided absolutely no post-baseline data. Patients achieving cCR who opt for Watch & Wait management are included in the mITT set for efficacy analysis.

### Sample size

Based on evidence of the results of previous clinical trials summarized in the systematic review, the pCR rate of nCRT alone is approximately 16% (5), and the pCR rate of nCRT combined with ICIs is approximately 36% (13). Therefore, this study sets the null hypothesis (H0) at P0 = 0.16 and alternative hypothesis (H1) at P1 = 0.36. Using a two-sided test with α = 0.05 and β = 0.2, and accounting for a 20% dropout rate, the calculated total sample size is 40 patients. Computations were performed using PASS 2021 v21.0.3.

### Follow-up

The follow-up methods include outpatient visits and telephone follow-ups. The follow-up schedule consists of assessments every 3 months for the first 2 years, followed by visits once every 6 months in the 3rd year, and subsequently, visits once every 1 year for the 4th–5th years. Procedure refer to ESMO Rectal Cancer Guidelines (2017) (10). Follow-up evaluations include, but are not limited to DRE, carcinoembryonic antigen measurements, endoscopy and biopsy, abdominal enhanced computed tomography, HRMRI, and anal cavity ultrasound.

## Discussion

Currently, China faces a substantial proportion of patients with mid-to-low LARC, emphasizing the importance of achieving R0 resection and preserving anal function. Neoadjuvant therapy has become the standard treatment for LARC, and the pCR rate of neoadjuvant long-course concurrent chemoradiotherapy alone is approximately 16% (5). Consequently, optimizing the neoadjuvant treatment approach for patients with LARC has emerged as a central scientific concern in this field.

In recent years, the application of the TNT mode has gained traction, particularly in patients with refractory LARC. Phase III randomized controlled clinical studies have reported that TNT mode elevates the pCR rate of patients with LARC to 28%, the organ function preservation rate to 50%, and reduced the distant metastasis rate to 17% (15). TNT offers the advantage of more substantial tumor shrinkage, a significant increase in the cCR rate, and more opportunities for ‘Watch and Wait’. This approach preserves the structure and function of organs, ultimately enhancing the quality of life for patients with LARC (16). Moreover, the 7-year follow-up results of the PRODIGE-23 study (7), presented at the 2023 ASCO Annual Meeting, highlighted that the TNT regimen, consisting of three-agent, high-intensity induction chemotherapy combined with long-course concurrent chemoradiotherapy, not only enhanced the pCR rate (28% *vs*. 13%) but also conferred a long-term survival benefit. However, 43% of the study’s patient population had T2–T3b stage and 13% had high location rectal cancer (10–15 cm from the anal verge). This raises a cautious consideration regarding the necessity of high-intensity preoperative neoadjuvant therapy for patients with LARC without high-risk factors. In response to this question, the OPRA study specifically enrolled only patients with low LARC (17). The results hinted that the TNT mode, comprising neoadjuvant long-course concurrent chemoradiotherapy plus sequential full cycles consolidation chemotherapy, improved the de-operation survival rate for patients. The CAO/ARO/AIO-12 study further affirmed that consolidation of full cycles of chemotherapy after long-course concurrent chemoradiotherapy produced a higher pCR rate than induction approach (18). The precise stratification of neoadjuvant therapy for patients with LARC has become a pivotal topic in clinical multidisciplinary consultations. Currently, ICIs has been demonstrated to be effective in dMMR/MSI-H LARC, avoiding the toxicity of nCRT and radical surgery (19). This underscores the importance of precise adjustments in neoadjuvant therapy for patients with LARC.

To further refine the stratification of patients with LARC, the RAPIDO study (8) enrolled patients with LARC with at least one high-risk factor (cT4, EMVI+, cN2, MRF+, and lateral lymph node+). The results demonstrated that a short course of radiotherapy combined with a full-cycles of neoadjuvant chemotherapy had advantages in increasing the pCR rate and reducing the 3-year treatment failure rate and distant conversion in the enrolled population. However, it is apparent from the reported data that the efficacy of tumor shrinkage with the TNT modality seems to have reached a plateau. Nevertheless, from a safety perspective, this modality appears feasible for the majority of patients with LARC and has received recommendations in clinical guidelines (4).

It is well known that radiation therapy helps to remodel the tumor immune microenvironment and exerts a potential synergistic effect on ICIs. On this basis, the VOLTAGE study first revealed that nCRT sequential Nivolumab could increase the pCR rate of LARC (12). More recently, four studies from China reported that adding PD-1 inhibitors during neoadjuvant therapy in LARC patients could further improve the pCR or cCR rates (20–23). However, current research has primarily focused on the combination modality of short-course radiotherapy and immunotherapy. Short-course radiotherapy (25 Gy/5 fractions) is disadvantaged by underdosing compared with long-course concurrent radiotherapy (50 - 50.4 Gy/25–28 fractions). Previous studies suggest that short-course radiotherapy lacks a significant downstaging effect, has a lower rate of anal preservation, and exhibits a higher rate of local recurrence compared with long-course concurrent chemoradiotherapy, especially in patients with lower margins are within 5 cm of the anal verge (24). Consequently, clinical guidelines for rectal cancer diagnosis and treatment, such as those from National Comprehensive Cancer Network, ESMO, and Chinese Society of Clinical Oncology, recommend long-course concurrent chemoradiotherapy for such patients. For patients with subperitoneal LARC, who are not initially resectable or face challenges in achieving R0 resection, and express a strong desire for anal preservation, further exploration is needed to assess the feasibility of full-cycles nCRT combined with immunotherapy.
